# Who is waiting to see the surgeon? sociodemographic, clinical characteristics and previous osteoarthritis care of people with knee osteoarthritis referred to public hospitals in Victoria, Australia

**DOI:** 10.1016/j.ocarto.2026.100818

**Published:** 2026-05-15

**Authors:** Juanita Low, Danilo De Oliveira Silva, Allison M. Ezzat, Alison J. Gibbs, Bernadette Brady, Michelle M. Dowsey, Vincent Lengkong, Adrienne Forsyth, Clarice Y Tang, Ilana N. Ackerman, Jason A. Wallis, MOTION Research Team, Christian J. Barton

**Affiliations:** a La Trobe Sports and Exercise Medicine Research Centre, La Trobe University, Bundoora, Australia; b School of Allied Health, Human Services and Sport, La Trobe University, Bundoora, Australia; c Department of Physical Therapy, Faculty of Medicine, University of British Columbia, Vancouver, BC, Canada; d Physiotherapy Department, Eastern Health, Box Hill Hospital, 8 Arnold Street, Box Hill, 3128, Victoria, Australia; e Liverpool Hospital, South Western Sydney Local Health District, Locked Bag 7103 Liverpool, BC, Sydney, NSW, 1871, Australia; f School of Health Sciences, Western Sydney University, Locked Bag 1797, Penrith, NSW, 2751, Australia; g Sydney School of Health Sciences, Faculty of Medicine and Health, The University of Sydney, Sydney, NSW, 2006, Australia; h Department of Orthopaedics, St Vincent's Hospital, Melbourne, Victoria, Australia; i University of Melboune Department of Surgery, St. Vincent's Hospital, Melbourne, Victoria, Australia; j School of Behavioural and Health Sciences, Australian Catholic University, Melbourne, Victoria, Australia; k Institute for Health and Sport, Victoria University, Melbourne, Australia; l Musculoskeletal Health and Wiser Health Care Units, School of Public Health and Preventive Medicine, Monash University, Melbourne, Victoria, Australia

**Keywords:** Knee, Osteoarthritis, Diversity, First-line care

## Abstract

**Aim:**

To describe the sociodemographic, clinical characteristics, and previous care received by people with knee osteoarthritis awaiting orthopaedic assessment, and compare these factors between culturally and linguistically diverse (CALD) participants and the wider population.

**Methods:**

318 people with knee osteoarthritis referred for orthopaedic assessment at four Australian public hospitals were included. CALD was defined as being born in a non-English-speaking country. Baseline variables included age, sex, education level, body mass index (BMI), worst and average knee pain (0–100 numeric rating scale), knee-related quality of life (Knee Injury and Osteoarthritis Outcome Score – Quality of Life [KOOS-QoL, 0–100]) subscale, self-reported health (EuroQoL Visual Analogue Scale [EQ-VAS], 0–100), physical activity participation (The University of California, Los Angeles [UCLA] Physical Activity Scale), radiographic severity (Kellgren-Lawrence grade), and previous osteoarthritis care (OsteoArthritis Quality Indicator Score v2: OA-QIv2, 0–100). Group comparisons were made using independent t-tests and proportion tests.

**Results:**

Participants presented with (mean ± SD): age (67 ± 10 years); BMI (32.2 ± 7.3 kg/m2); knee pain (worst:74 ± 22 mm; average:53 ± 22 mm); KOOS-QoL (30 ± 19); EQ-VAS (63 ± 20 mm); and OA–QIv2 (49 ± 23). 52% had completed tertiary/vocational degrees. Compared with the wider population, CALD background participants (n = 69, 32 countries) were older (mean difference 5 years, 95%CI = 2 to 7), had lower BMI (−3.6 kg/m2, 95%CI = −5.5 to −1.7), and a smaller proportion had tertiary education (difference = −14%, 95%CI = −27% to −1%). Clinical characteristics were similar between groups.

**Conclusions:**

Regardless of CALD background, people with knee osteoarthritis in Australia typically present with clinical and radiographic symptoms that may warrant orthopaedic referral, yet may not receive optimal osteoarthritis care beforehand.

## 1 Introduction

Knee osteoarthritis is a leading cause of chronic pain and disability worldwide, resulting in high economic burden to health systems [1]. International guidelines consistently recommend first-line non-surgical care (exercise, education, and weight management, when indicated) for all people with knee osteoarthritis, while second-line passive and pharmacological treatment recommendations are less consistent [2]. Knee replacement (TKR) surgery is recommended only for those with moderate-severe osteoarthritis who have adequately trialled non-surgical care [3]. However, evidence from around the world indicates that a large proportion of people with knee osteoarthritis do not adequately access first-line care prior to referral for orthopaedic assessment [[4], [5], [6], [7], [8]].

Commonly reported sociodemographic and clinical characteristics such as education, sex, body mass, age, and self-reported disability are associated with access to surgical and non-surgical care in people with knee osteoarthritis [9]. Older age, higher BMI, and lower education level are reported to be associated with reduced healthcare access and negative perioperative outcomes [[10], [11], [12]]. Sociodemographic characteristics related to ethnic diversity (e.g. nationality, country of birth, culture and language) are less commonly reported [9]. Previous research reports that ethnic minorities report lower TKR utilisation rates, longer lengths of stay after undergoing surgery, higher complication rates, worse pain, and poorer functional outcomes post-TKR [[13], [14], [15]]. However, other than in the United States, less is known about whether access to guideline-recommended osteoarthritis care prior to surgical consideration varies with ethnic diversity [16].

In Australia, sociodemographic characteristics related to ethnic diversity are described using the term “Culturally and Linguistically Diverse” (CALD), and include characteristics such as country of birth and language spoken [17]. Previous research evaluating an osteoarthritis management program in Australia has reported that having a CALD background may be a barrier to accessing first-line care for people with knee osteoarthritis [18]. However, contemporary knowledge regarding what care has been received by people with knee osteoarthritis in Australia prior to referral for orthopaedic assessment is scarce, including whether prior care may differ by CALD backgrounds [18].

Health systems are becoming overwhelmed with rapidly escalating costs and long waitlists for hospital-based orthopaedic assessment and TKR surgeries [19]. Understanding sociodemographic (including CALD characteristics) and clinical characteristics of people with knee osteoarthritis referred for hospital-based orthopaedic assessment may highlight gaps in healthcare provision. This knowledge could inform initiatives to address long surgical waitlists, enhance healthcare efficiency, and improve treatment outcomes and satisfaction. Therefore, our aims were to: (i) describe the sociodemographic (including CALD characteristics), clinical characteristics, and previous osteoarthritis care received by people with knee osteoarthritis referred for public hospital-based orthopaedic assessment in Victoria, Australia; and (ii) to explore differences in sociodemographic, clinical characteristics and previous osteoarthritis care received between people characterised as CALD and the wider population.

## 2 Methods

### 2.1 Design

This cross-sectional study used baseline data from participants who were part of a pre-registered (MonitOring The Influence Of care for patients with kNee osteoarthritis [MOTION]), parallel hybrid cohort-implementation clinical trial study (St Vincent's Hospital, Melbourne Human Research Ethics Committee reference: 251/21) [20]. Our findings were reported according to Strengthening the Reporting of OBservational studies in Epidemiology (Appendix A) [21].

### 2.2 Participants

Eligible participants, regardless of their preferred speaking language, were referred by a general practitioner (GP) for orthopaedic assessment to one of four metropolitan or regional hospitals in Victoria, Australia, between May 2022 and February 2024, with a clinical diagnosis of knee osteoarthritis, and had available knee imaging. Participants were ineligible if they had: (i) been waitlisted for urgent orthopaedic assessment; (ii) a primary musculoskeletal complaint that was not knee osteoarthritis (e.g., low back pain); (iii) completed a structured education and exercise program in the last 12 months; (iv) a history of any rheumatological conditions (rheumatoid arthritis, fibromyalgia etc.), neurological conditions and/or cognitive impairments; (v) received medical advice prohibiting exercise; (vi) previous knee joint replacement surgery on the symptomatic knee (vii) previous osteotomy or artificial (e.g., metal) implants associated with the affected joint in situ; or (viii) received any orthopaedic or neurological surgery to the lower limb or low back in the past year. In this pragmatic study, we aimed to recruit as many participants as possible over an 18-month period. Our participant sample size was informed by pilot data from participating hospital sites, and details were reported previously [20].

### 2.3 Procedures

Participants were contacted and screened by phone, by trained research assistants. Eligible participants were provided with verbal information during the phone call, and a written information statement and consent form was posted to them if they were interested. They were given the opportunity to discuss the study with their GP and/or family and friends before returning a written signed consent form via post. After receiving written consent, a baseline phone interview was conducted with all participants to collect their self-reported sociodemographic variables including height, body mass, age, sex, education levels (Non-tertiary or Tertiary/vocational), and CALD-related characteristics (including country of birth, preferred-spoken language, and self-rated English proficiency [Speaks well or Does not speak well]). This data was entered directly into the secure online Research Electronic Data Capture (REDCap) system by the research assistant. Body mass index (BMI, kg/m^2^) was calculated from self-reported height and body mass. Additionally, baseline clinical characteristics were obtained from patient-reported outcome measures (outlined below) collected during the phone interview, and entered directly into REDCap.

### 2.4 Characterising participants from CALD backgrounds, and data collection from participants with limited English proficiency

We have adopted the term ‘CALD’ to describe participants who self-reported being born in a non-English-speaking country [17]. While we recognise that this definition is somewhat narrow and may not fully capture the rich diversity and complexity encompassed by the umbrella term ‘CALD’, it aligns with the classifications used by the Standard Australian Classification of Countries for the Census [22], facilitating consistency and comparability with existing research. Participants who are not considered as ‘CALD’ based on classifications used by the Standard Australian Classification of Countries for the Census [22] are defined as ‘the wider population’ in this study. Additionally, we included other CALD-related factors (e.g., speaking language other than English, self-rated English proficiency) recommended by Australian Standards for Statistics on Cultural and Language Diversity [23] to further describe our participants.

For participants preferring a non-English language of communication and were unable to speak English well, patient-reported outcome measures were completed either by (i) a bilingual study team member (JL) using validated translated versions in Chinese, or (ii) by study team members through interpreter proxies (e.g. participants’ self-nominated caregivers/family members) using English questionnaires. The use of interpreter proxies is a reliable alternative to professional healthcare interpreters in Australia when collecting patient-reported outcome measures and was a deliberate strategy to maximise inclusivity and accessibility [24].

### 2.5 Clinical characteristics

#### 2.5.1 Knee pain intensity

Worst and average knee pain intensities over the past month were measured using a 100-point numeric rating scale (NRS: 0 = “no pain at all” and 100 = “worst imaginable pain”). The numerical rating scale is reliable, valid, responsive, and a frequently used pain outcome measure for knee osteoarthritis (ICC 95% CI = 0.95 (0.93–0.96) [25].

#### 2.5.2 Knee-related quality of life

The Knee Injury and Osteoarthritis Outcome Score – Quality of Life (KOOS-QoL) subscale was used to assess knee-related quality of life (QoL). The KOOS-QoL subscale is frequently used as an outcome measure for knee osteoarthritis as it is reliable, valid, and responsive (ICC = 0.89) [26]. Comprising of 4 items scored from 0 to 4 on a Likert scale, scores obtained were transformed to a 0 (extreme knee problems) - 100 (no knee problems) scale.

#### 2.5.3 Self-reported health

The EuroQol visual analogue scale (EQ-VAS) is a self-reported global assessment of health and was used as a measurement of health-related QoL [27]. It has been found to have sufficient construct validity and reliability with estimating health-related QoL [27]. The EQ-VAS was anchored with 0 (worst possible health) to 100 (best possible health).

#### 2.5.4 Physical activity participation

The University of California Los Angeles (UCLA) Physical Activity Scale was used to measure physical activity participation [28]. The UCLA physical activity scale has construct validity and reliability (Kappa coefficient = 0.80–0.86), and is a recommended scale for evaluating self-reported physical activity participation in osteoarthritis populations [28].

The UCLA physical activity scale measures physical participation on a 10-point scale, ranging from 1 “Wholly inactive: dependent on others; cannot leave residence” to 10 “Regularly participates in impact sports such as jogging, tennis, skiing, acrobatics, ballet, heavy labour, or backpacking”. Physical activity participation data collected were further categorised as ‘high’ (≥7), ‘moderate’ (5–6) or ‘low’ activity levels (≤4) [28].

#### 2.5.5 Radiographic severity of osteoarthritis

The Kellgren-Lawrence system was used to classify the radiographic severity of participants' knee osteoarthritis with five grades ranging from 0 (none) to 4 (severe) [29]. Kellgren-Lawrence Classification system is valid, reliable, and is the most widely used clinical tool to diagnose and assess the severity of knee osteoarthritis radiographically [29]. Kellgren-Lawrence grades (K-L grade) were then further categorised to ‘moderate-severe’ (≥3) or ‘none-mild’ (≤2) groups [30]. Usual practice in Victoria requires GPs to provide radiographs in weightbearing anterior-posterior, Rosenburg, skyline, and lateral views when referring patients to public orthopaedic services, but a specific protocol was not enforced for this study [31]. Therefore, to standardise the radiographic grading assessment, all X-rays taken within 12 months were obtained from participants' referrals and graded by an advanced practice musculoskeletal physiotherapist who has had extensive training and experience in clinical and radiological assessment of patients with knee osteoarthritis [32].

#### 2.5.6 Previous osteoarthritis care received

The OsteoArthritis Quality Indicator questionnaire v2 (OA-QIv2) was used to assess the quality of osteoarthritis care participants had received in the past 12 months, across domains of patient education and information about osteoarthritis, clinician assessments, referrals, and pharmacological treatment [33]. The 16-item OA-QIv2 is reliable, valid, and responsive (Test-retest reliability for participants who had not received osteoarthritis intervention over 2-weeks, ICC 95% CI = 0.89 (0.83–0.93) [33]. An item was considered “eligible” with either a “Yes” or “No” response; a “Yes” response reflected achievement of the item. The number of quality indicator items achieved (i.e., total pass rates) with each participant was calculated and expressed as a percentage [(total number of items with a “Yes” response)/(the number of eligible items), 0–100%]. A higher total pass rate implies better quality of osteoarthritis care received. Group pass rate ([Total number of “Yes” response to item]/[Total eligible responses to item], 0–100%) was calculated for all response items, and referrals to first-line care health professionals was measured using two questions: OA-QIv2 items 5 (referral to exercise) and 7 (referral to weight management). Second-line care was assessed with items 12 to 14 (medications), and 15 (joint injection). Quality indicator items that participants self-reported as not applicable were captured by a third response option (e.g., item 7: “Not overweight”; Appendix B), and not factored into group pass rate calculation.

### 2.6 Statistical analysis

Baseline sociodemographic and clinical characteristics were summarised descriptively for the full sample, and for CALD and the wider population. Categorical variables (sex, country of birth, education level, language, self-rated English proficiency, physical activity participation level, and radiographic severity of osteoarthritis) were reported as frequencies and percentages. Continuous variables (age, BMI, knee pain intensity, knee-related quality of life, self-reported health, and previous osteoarthritis care received) were reported as means and standard deviations.

Sociodemographic, clinical characteristics, and previous osteoarthritis care received were compared between CALD and the wider population using the Welch Two Sample *t*-test for continuous variables. Two-sample test of proportions was used for all categorical variables. Our analyses were descriptive and exploratory; no multivariable modelling was undertaken as the study was not designed to evaluate independent predictors or causal associations. Statistical analysis was performed using R software, version 4.3.3.

## 3 Results

### 3.1 Participants

**Fig. 1:**
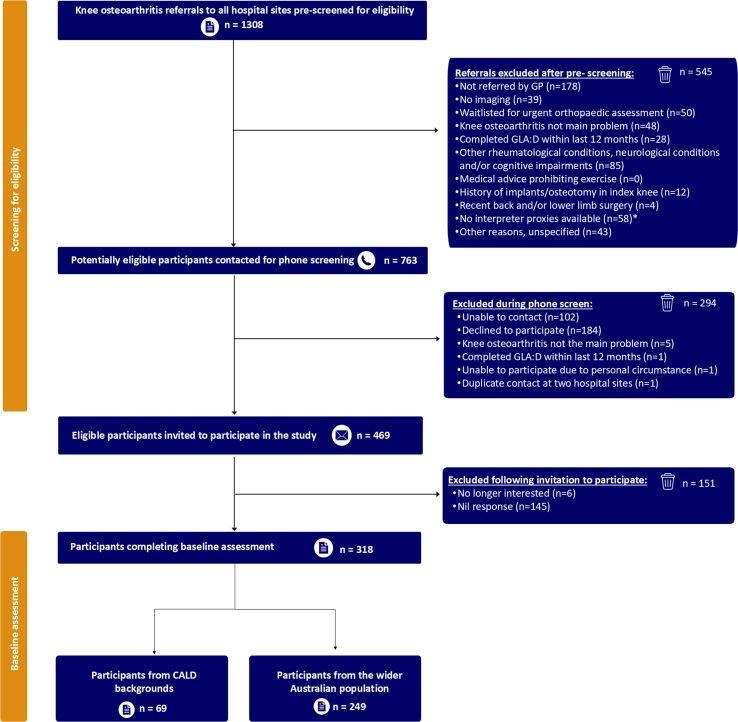
Recruitment flowchart.

A total of 1308 individuals were screened for eligibility across four public hospital sites, with 763 identified as potentially eligible and 469 able to be contacted and invited for this study, which resulted in 318 included (Fig. 1). Included participants had a mean age of 67 (standard deviation (SD) = 10 years) and a mean BMI of 32.2 kg/m2 (SD 7.3kg/m2). Fifty-seven percent were female, and 52% had obtained tertiary education or vocational training (Table 1). Overall, 23% (n = 74) had none-mild (K-L grade 0–2) radiographic osteoarthritis and 69% (n = 220) had moderate-severe (K-L grade 3–4) radiographic osteoarthritis (Table 3). Participants of CALD backgrounds made up 22% (n = 69) of the total cohort. Thirty-two countries were represented by participants of CALD backgrounds (n = 69), and six by the wider population (n = 249), of which, 49% were from the Asia-Pacific region (Appendix C). Twenty-four different non-English languages were spoken by participants of CALD backgrounds (Appendix D). Twenty participants of CALD backgrounds reported that they did not speak English well (Table 2); four completed both KOOS-QoL and EQ-VAS using the Chinese questionnaires (facilitated by bilingual team member JL), and 16 completed the English questionnaires via interpreter proxies.

**Table 1:** Summary of sociodemographic details of people with knee osteoarthritis referred to public hospitals in Australia^a^.

Sociodemographic variable	Overall cohort (n = 318)	CALD participants^b^ (n = 69)	Wider population participants (n = 249)	Between group comparison Mean/Proportion difference (95% CI)
**Age, years, mean ± SD**	67 ± 10	71 ± 9	66 ± 10	**5 (2–7)**
**BMI, kg/m^2^, mean ± SD**	32.2 ± 7.3	29.4 ± 6.0	33.0 ± 7.4	**−3.6 (−5.5− −1.7)**
**Sex, % female (n)**	57% (182)	61% (42)	56% (140)	5% (−8%–2%)
**Educational level, % tertiary/Vocational (n)**	52% (164)	41% (28)	55% (136)	**−14%(−27%–** −**1%)**
**Country of birth, % not Australia (n)**^b^	35% (112)	100% (69)	17% (43)	–

a Statistically significant between-group differences are shown in bold.

b We characterised participants born in a non-English-speaking country of birth as CALD.

**Table 2:** Summary of CALD-related sociodemographic factors in CALD participants^a^.

CALD-related factor	CALD participants^a^ (n = 69)
**Country of birth, % not Australia (n)**	100% (69)
**Speaks language other than English, % yes (n)**	72% (50)
**Self-rated English proficiency** [18]**, % does not speak well (n)**	29% (20)

a We characterised participants born in a non-English-speaking country of birth as CALD.

**Table 3:** Summary of clinical characteristics and quality of previous osteoarthritis care of people with knee osteoarthritis referred to public hospitals in Australia.

Clinical characteristic	Overall cohort (n = 318)	CALD participants (n = 69)	Wider population participants (n = 249)	Between-group comparison
				Mean/Proportion difference (95% CI)
**Worst knee pain- NRS, mean** ± **SD (range)**	74 ± 22 (8–100)	74 ± 22 (9–100)	74 ± 22 (8–100)	0.4 (−5–6)
**Average knee pain- NRS, mean** ± **SD (range)**	53 ± 22 (0–100)	56 ± 20 (2–97)	52 ± 22 (0–100)	4 (−2–10)
**KOOS-QoL, 0**–**100, mean** ± **SD (range)**	30 ± 19 (0–81)	30 ± 21 (0–81)	30 ± 18 (0–75)	0.2 (−5–5)
**EQ-VAS, mm, mean** ± **SD (range)**	63 ± 20 (5–100)	61 ± 20 (5–95)	64 ± 19 (7–100)	−3 (−8–3)
**OA-QIv2 total pass rate**^a^**, 0–100%, mean** ± **SD (range)**	49 ± 23 (0–100)	50 ± 22 (0–100)	49 ± 23 (0–100)	1 (−5–7)
**Physical activity participation level, % (n)**				
UCLA “high”	11% (34)	9% (6)	11% (28)	−2% (−10%–5%)
UCLA “moderate”	31% (98)	28% (19)	32% (79)	−4% (−16%–8%)
UCLA “low”	59% (186)	64% (44)	57% (142)	7% (−6%–20%)
**Kellgren-lawrence grade, % (n)**				
None-mild	23% (74)	29% (20)	22% (54)	7% (−5–19%)
Moderate-severe	69% (220)	65% (45)	70% (175)	−5% (−18%–8%)
X-rays unavailable for grading	8% (24)	6% (4)	8% (20)	−2% (−9%–4%)

a The OsteoArthritis OsteoArthritis Quality Indicator questionnaire v2 (OA-QIv2) total pass rate with each participant was calculated and expressed as a percentage [(total number of items with a “Yes” response)/(the number of eligible items)]. A higher total pass rate implies better quality of osteoarthritis care received.

### 3.2 Sociodemographic and clinical characteristics of participants of CALD backgrounds

CALD participants (n = 69) were older (mean difference 5 years, 95%CI = 2 to 7), had lower BMI (−3.6 kg/m2, 95%CI = −5.5 to −1.7), and less frequently had tertiary education (14% fewer; 95%CI = −27% to −1%) (Table 1). Group differences between CALD and the wider population across sex, knee pain intensity, knee-related QoL, health-related QoL, physical activity participation, radiographic severity of osteoarthritis, and quality of previous osteoarthritis care received were small (Table 1, Table 3).

### 3.3 Previous osteoarthritis care received

The overall mean total OA-QIv2 pass rate was 49% (SD ± 23), with no difference between CALD and the wider population (Table 3). Individual group pass rates for items of OA-QIv2 for all participants, CALD, and the wider population, are reported in Table 4. Fifty-one percent of participants were referred to an exercise professional (item 5), and 31% were referred for weight management (item 7). Medication use (items 12, 13, 14) was reported by 51%–74%, and 39% reported being offered a joint injection (item 15). Compared to participants from the wider population, 19% more CALD participants self-reported being not overweight (Appendix B, item 7).

**Table 4:** Summary of osteoarthritis care received, OsteoArthritis Quality Indicator Questionnaire v2 (OA-QIv2)^a^.

Group pass rate for all eligible participants for each item, % yes (n)	Overall cohort	CALD participants	Wider population participants	Proportion Difference^b^ (95% CI)
*1. Have you been given information on osteoarthritis from a health professional?*	42% (126)	43% (27)	42% (99)	1% (−12%–15%)
*2. Have you been given information about different treatment alternatives?*	34% (103)	30% (19)	34% (84)	−4% (−17%–9%)
*3. Have you been given information about how you can self-manage the disease?*	31% (95)	27% (17)	32% (78)	−5% (−17%–8%)
*4. Have you been given information about the importance of physical activity and exercise?*	57% (178)	57% (38)	57% (140)	0% (−14%–13%)
*5. Have you been referred or offered a referral to a health professional who can advise you about physical activity and exercise?*	51% (160)	55% (37)	49% (123)	6% (−8%–19%)
*6. Have you been advised to lose weight, if you are overweight?*	68% (140)	68% (23)	68% (117)	0% (−18%–16%)
*7. Have you been referred or offered a referral to someone who can help you to lose weight, if you are overweight?*	31% (63)	33% (11)	31% (52)	2% (−15%–20%)
*8. If you have problems with daily activities, have these problems been assessed by a health professional?*	19% (39)	26% (12)	17% (27)	9% (−5%–22%)
*9. If you have problems with walking, has your need for a walking aid been assessed? (*e.g. *stick, crutch or walker)*	21% (39)	34% (15)	17% (24)	18% (2%–33%)
*10. If you have problems related to other daily activities, has your need for appliances and aids been assessed? (e.g.splints, assistive technology for cooking or personal hygiene, a special chair)*	17% (31)	24% (10)	15% (21)	9% (−5%–23%)
*11. If you have joint pain, has it been assessed by a health professional?*	76% (239)	72% (50)	77% (189)	−4% (−16%–8%)
*12. If you have joint pain, was paracetamol the first medication that was recommended?*	74% (232)	82% (55)	72% (177)	10% (−1%–21%)
*13. If you have prolonged severe joint pain, which is not relieved sufficiently by paracetamol, have you been offered stronger pain killing medication? (e.g co-codamol,codeine, tramadol, co-proxamol, co-dydramol,dihydrocodeine)*	51% (124)	46% (26)	53% (98)	−7% (−22%–7%)
*14. If you use anti-inflammatory medications, have you been given information about the effects and possible side-effects of this medication? (e.g ibuprofen (Nurofen, Brufen), diclofenac (Voltarol), naproxen (Naprosyn), celecoxib (Celebrex))*	55% (116)	48% (25)	58% (91)	−10% (−25%–6%)
*15. If you have experienced an acute deterioration of your symptoms, have you been given or offered a steroid injection?*	39% (84)	50% (25)	36% (59)	14% (−1%–30%)
*16. If you are severely troubled by your osteoarthritis, and exercise and medication do not help, have you been referred or offered a referral for an assessment for operation? (e.g joint replacement)*	90% (253)	89% (54)	90% (199)	−1% (−11%–7%)

a OA-QIv2 items 5 and 7 were used to assess for referrals made to other health professionals who are able to provide first-line care (i.e., exercise and weight management). Second-line care was assessed with items 12 to 14 (medications), and 15 (joint injection).

b A negative difference indicates a higher percentage for participant group of the wider population.

## 4 Discussion

Of the 318 participants with knee osteoarthritis referred for orthopaedic assessment at Australian public hospitals in our study, 22% were from CALD backgrounds. Both participants from CALD and the wider population typically presented with high knee pain intensity, low knee-related QoL, and moderate-severe radiographic osteoarthritis. Based on mean total pass rates of the osteoarthritis quality indicators (OA-QIv2), our cohort reported that only half the quality indicator items were achieved, which is similar to previous European-based cohorts [34]. Compared to participants of the wider population, CALD participants had lower BMI, were older, and fewer had received tertiary education/vocational training. However, group differences between CALD and the wider population across clinical characteristics and quality of care were small and unlikely to be clinically meaningful. This was somewhat surprising considering that CALD populations have been reported to have greater and additional barriers to accessing healthcare compared to the wider population in other research [18,35]. It suggests that inefficiencies in first-line osteoarthritis management before orthopaedic referral may be systemic, regardless of CALD background. Developing and testing strategies to strengthen universal access to appropriate non-surgical care, including education and exercise therapy, prior to surgical assessment is therefore strongly encouraged for all people with knee osteoarthritis. This is essential to reduce inappropriate referrals for surgical assessment, and to reduce surgical waitlist pressures, with possible options at both health system (e.g. improved funding) and health services (e.g. implementing new services) levels.

### 4.1 Baseline sociodemographic, clinical characteristics and previous osteoarthritis care

Our participants reflect the diverse cultural and linguistic backgrounds of the broader Australian population [17,36], with 35% born outside of Australia (Australia-wide: 31%), and 16% speaking a language other than English (Australia-wide: 23%) [17,36]. The proportion of participants reporting the completion of tertiary or vocational training (52%) was also similar to the broader Australian population (Australia-wide, 65–74 years: 57%) [37]. Consistent with cohorts referred for orthopaedic assessment at public hospitals in New South Wales, Queensland, and Victoria, Australia [[38], [39], [40]], and other large knee osteoarthritis cohort studies [18,41], including the Good Life with osteoArthritis in Denmark (GLA:D®) Australia registry [18], our participants were predominantly female, were on average 67 years old, and the mean BMI indicated that they were living with obesity or overweight [42]. Our cohort had a lower proportion of non-English speakers (6%) comparted to the cohort in New South Wales (10%) [38]. Other cohorts did not report English-language proficiency-related data [39,40], or excluded non-English speakers [18,41].

Our cohort had, on average, moderate-to-high knee pain severity, poor knee-related QoL, and majority had moderate-to-severe radiographic knee osteoarthritis, which are minimum clinical requirements to justify an orthopaedic referral for TKR [3]. However, it is notable that: (i) the cohort was on average living with obesity; (ii) many reported that they were not referred for exercise or weight management; (iii) more than half (n = 186) reported low levels of physical activity participation; (iv) almost a quarter (n = 74) were assessed to have none to mild radiographic knee osteoarthritis; and (v) there was variability in self-reported clinical characteristics, indicating that some participants experienced milder levels of clinical severity. These findings highlight that while most referrals for orthopaedic assessment appear justified, some may not be (e.g., low radiographic and clinical severity), and there are potential opportunities for better symptom management through engagement with non-surgical first-line care (exercise, education, weight management), regardless of radiographic or clinical severity, before orthopaedic assessment [2].

Low rates of prior referral to exercise (51%, n = 160) and weight management (31%, n = 63) reported by our cohort reflects a global trend where many people with osteoarthritis do not receive adequate guideline recommended first-line care prior referral for orthopaedic assessment [4,7,8,43]. Improving the uptake of first-line care prior to orthopaedic referral can facilitate more efficient triaging and may save the health system substantial money. For example, a previous budget impact analysis in Australia reported that providing access to first-line care for all people with moderate-severe knee osteoarthritis could result in an estimated cost-savings of $AUD303-690 million annually for the healthcare system [44]. More recent analysis indicates that ensuring education and exercise therapy is adequately trialled prior to TKR may be cost effective over a 9-year time horizon, and cost effective over the lifetime in those with low to mild pain [45]. Given the wide range of clinical severity in our participants, strategies that improve the uptake of first-line care before orthopaedic assessment are urgently needed to optimise clinical outcomes and improve healthcare efficiency.

Beyond first-line care, our findings highlight discrepancies between clinical practice and higher-quality guidelines related to second-line passive and pharmacological interventions [2]. Almost three-quarters of our cohort reported that paracetamol was the first medication recommended for their knee pain, which may be influenced by historical guideline recommendations supporting this [46]. However, it should be acknowledged that there is a lack of evidence of efficacy for paracetamol [47], and more recent guidelines provide more conflicting recommendations [2]. We also found that approximately half of our participants with self-reported prolonged, severe knee pain were offered opioids when paracetamol did not provide sufficient pain relief. This is concerning as guidelines recommend against the use of strong opioids due to risk of adverse events and minimal benefits for pain [2]. Next, of those using non-steroidal anti-inflammatory drugs, only half reported that they were informed about the associated safety risks [2]. Considering the potential harms of non-steroidal anti-inflammatory drugs [48], this apparent lack of education should be addressed. Additionally, there were low reported rates of joint injections (39%) in those who had experienced an acute deterioration of symptoms. This may reflect concerns about cartilage degradation [49] despite consistent guideline recommendations for steroid injections in these instances [2]. The varied use of second-line management for knee osteoarthritis, and common misalignment of this practice with guideline recommendations, identified in our study could stem from the lack of clarity, trust, and awareness of clinical practice guideline recommendations [2].

### 4.2 Differences between CALD participants and participants of the wider population with knee osteoarthritis, and previous osteoarthritis care received

Based on self-reported data, CALD participants had a lower average BMI, and fewer were overweight based on commonly accepted definitions compared to participants of the wider population [42]. While these results may suggest that CALD participants are less likely to require access to weight management services, it is important to note that BMI classification and cut-points are based off White, Hispanic, and Black populations [42]. Although commonly used, they may not be reflective of the diversity in our CALD sample where almost half were born in Asia-Pacific countries. Notably, cut-points for the Asia-Pacific populations are 23–24.9 kg/m2 for the overweight category and ≥25 kg/m2 for obese category [50]. Thus, using a standard BMI classification scale with CALD individuals may underestimate the number of people living with obesity, and if followed clinically, result in less access to weight management services, despite these being warranted. When providing care to CALD people, clinicians should take their diverse background into consideration during clinical decision-making to ensure equitable access to weight management when needed.

Our findings that CALD participants were older and had lower education attainment than participants of the wider population are notable, with both factors associated with lower health literacy [51]. Lower health literacy, combined with potential cross-cultural communication challenges, can hinder understanding of medical information and engagement with healthcare services [52]. Healthcare providers should be equipped with the skills to adapt communication strategies (e.g., pictorial guides as education material, translators, support groups) to meet the diverse needs of CALD patients. Involving diverse communities in the development of educational materials and care pathways can further enhance the accessibility and effectiveness of osteoarthritis care [53].

### 4.3 Strengths and limitations

Of 469 eligible participants identified, 318 (68%) consented to participate. While this was similar to another study recruiting people with knee osteoarthritis from an Australian public health system [54], limited reporting in other similar Australian studies, including their screening processes, prevented us from assessing whether our recruitment rate reflects other similar populations [[38], [39], [40],^55^]. For transparency and to inform future research in public hospital settings, we have reported our recruitment and response rates at all stages of recruitment. Without consent to collect data from non-consenting people, we are unable to determine whether they might differ systematically in clinical severity, sociodemographic characteristics, or CALD-related status compared with those who consented to participate. This introduces potential selection bias and may limit the generalisability of our findings. We attempted to reduce selection bias by recruiting participants across four metropolitan and regional hospital sites, and applying broad eligibility criteria (e.g., no language restrictions), resulting in a proportion of participants from CALD backgrounds comparable to that of the Australian population [17,36]. While those declining did not disclose specific reasons, possible reasons include time-cost for the participants, lack of financial incentives, and fears regarding data security. Without consent for data collection and/or use from those who declined participation, we are unable to compare their sociodemographic diversity with those participating to understand if other under-represented groups were missed. We used CALD indicators recommended by the Australian Bureau of Statistics (e.g., country of birth) to characterise and contextualise our cohort, including ethnic diversity. This allows direct comparison to the broader Australian population, facilitating greater generalisability of our findings. However, we acknowledge that CALD is a broad classification which may not comprehensively represent the ethnic heterogeneity of our participants from CALD backgrounds [56]. Furthermore, we used language and place of birth to characterise our participants from CALD backgrounds, instead of using them as proxy measures to provide insights into the influence of acculturation on healthcare access [57]. Our population was recruited from public hospitals and may not reflect findings of those who sought care privately, where 70% of TKRs occur in Australia [58].

A specific imaging protocol was not enforced for this study, with imaging obtained from various radiology providers. However, all X-rays were assessed by one assessor to ensure consistency in K-L grading. Participants who had completed a structured education and exercise program in the past 12 months were excluded to avoid confounding the larger MOTION study [20] comparing usual hospital-based care with community-based care that includes structured education and exercise therapy. Without consent, we were unable to explore whether their baseline clinical outcomes or care received differed from included participants. However, only 2% (n = 29) of those screened for eligibility (n = 1308) were excluded, meaning this criterion is unlikely to substantially affect our OA-QIv2 findings. Our findings indicate varied responses to OA-QIv2, question 16 (Appendix B), with 9% (n = 28) reporting they had not been referred or offered a referral for an assessment for operation. It is possible that this number could be greater or smaller, as GP referrals for orthopaedic assessment (required for participation in this study) in the Victorian public health system are triaged to either an orthopaedic surgeon or a physiotherapy-led orthopaedic service [59]. Therefore, participants were unaware of who they might see for their orthopaedic assessment until they receive their initial appointment. The OA-QIv2, despite being used widely [33,34,43], has not been validated against objective measures of care delivery [33]. As responses are self-reported, findings reflect participant perception of care received, but may not accurately reflect actual care received, potentially leading to over- or underestimation.

Our data related to clinical characteristics (e.g., height, body mass, physical activity levels) and osteoarthritis care received are self-reported, which may be influenced by recall and social desirability bias. The UCLA physical activity scale is a single-item instrument and may not be sensitive at capturing meaningful interpretations of physical function, compared to other comprehensive questionnaires (e.g. KOOS Function in daily living or Western Ontario McMaster Universities Osteoarthritis Index Function subscales). However, we did not include them to minimise participant burden. Next, we used BMI alone to measure obesity, which, without anthropometric measures (e.g. waist circumference, waist-to-hip ratio), may not provide an accurate estimation of adiposity that impacts an individual's health [60]. Lastly, we acknowledge that not all translated patient-reported outcome measures have undergone validation. When validated translations were unavailable, data from non-English speaking participants were obtained with the assistance of bilingual study team members and/or family members/caregivers. While possible misinterpretation of items on patient-reported outcome measures may occur, this was a pragmatic approach taken to facilitate inclusion of participants reflective of the ethnic diversity in Australia and those underrepresented in research.

## 5 Conclusion

People with knee osteoarthritis referred for orthopaedic assessment in the Australian public health system are culturally and linguistically diverse, and present with clinical and radiographic symptoms that may justify referral for orthopaedic assessment. However, both CALD participants and participants of the wider population report receiving average-quality osteoarthritis care overall, with our findings suggesting that non-operative management may not be adequately trialled prior to referral in many cases. Improving access and uptake to high-quality, non-surgical knee osteoarthritis care before referral for orthopaedic assessment is needed to better align care provided with guideline recommendations. With nearly a quarter of participants from CALD backgrounds, healthcare systems must accommodate language and cultural diversity and consider sociodemographic differences when providing osteoarthritis care.

## Author contributions

JL, DOS, AME and CJB led and conceived the study design, with input from AJG, BB, MMD, VL, AF, CT, INA, JW. JL, VL and the MOTION research team collected the data for analysis. JL led the preparation of the manuscript, with input from CJB, DOS, AME. JL and CJB led the statistical analysis. All authors contributed to the drafting of the manuscript and approved the final version including of the manuscript.

## Ethics

The procedures followed were in accordance with the ethical standards of the responsible committee on human experimentation (institutional and national) and with the Helsinki Declaration of 1975, as revised in 2000.

## Role of funding source

This work was supported by the Medical Research Future Fund from the National Health and Medical Research Council (NHMRC) grant number [APP1175374] and the La Trobe University Medical Research Future Fund Kickstarter Scheme. DOS is a recipient of a National Health and Medical Research Council (NHMRC) of Australia Investigator Grant (Grant number: GNT2033417). AME was supported by a Canadian Institutes for Health Research Post-Doctoral Fellowship (2020–2023) and a Micheal Smith Health Research British Columbia Health Professional Investigator Award (2024–2029). MMD is the recipient of a University of Melbourne Dame Kate Campbell Fellowship.

## Conflict of interest

None declared.

## Figures and Tables

**Table:** 

	Item No	Recommendation	Location reported
**Title and abstract**	1	(*a*) Indicate the study's design with a commonly used term in the title or the abstract	Title page document
		(*b*) Provide in the abstract an informative and balanced summary of what was done and what was found	Title page document
**Introduction**			
Background/rationale	2	Explain the scientific background and rationale for the investigation being reported	L2 to L43
Objectives	3	State specific objectives, including any prespecified hypotheses	L38 to L43
**Methods**			
Study design	4	Present key elements of study design early in the paper	L54 to L67
Setting	5	Describe the setting, locations, and relevant dates, including periods of recruitment, exposure, follow-up, and data collection	L54 to L67
Participants	6	(*a*) Give the eligibility criteria, and the sources and methods of selection of participants	L54 to L103
Variables	7	Clearly define all outcomes, exposures, predictors, potential confounders, and effect modifiers. Give diagnostic criteria, if applicable	L85 to L103, L106 to L165
Data sources/measurement	8∗	For each variable of interest, give sources of data and details of methods of assessment (measurement). Describe comparability of assessment methods if there is more than one group	L106 to L165
Bias	9	Describe any efforts to address potential sources of bias	NA
Study size	10	Explain how the study size was arrived at	L66, study registration
Quantitative variables	11	Explain how quantitative variables were handled in the analyses. If applicable, describe which groupings were chosen and why	L106 to L179
Statistical methods	12	(*a*) Describe all statistical methods, including those used to control for confounding	L167 to L179
		(*b*) Describe any methods used to examine subgroups and interactions	L176- L179
		(*c*) Explain how missing data were addressed	NA
		(*d*) If applicable, describe analytical methods taking account of sampling strategy	NA
		(*e*) Describe any sensitivity analyses	NA
**Results**			
Participants	13∗	(a) Report numbers of individuals at each stage of study—eg numbers potentially eligible, examined for eligibility, confirmed eligible, included in the study, completing follow-up, and analysed	L182 to L195, Fig. 1
		(b) Give reasons for non-participation at each stage	Fig. 1
		(c) Consider use of a flow diagram	
Descriptive data	14∗	(a) Give characteristics of study participants (eg demographic, clinical, social) and information on exposures and potential confounders	L199 to L216, Table 1, Table 2
		(b) Indicate number of participants with missing data for each variable of interest	NA
Outcome data	15∗	Report numbers of outcome events or summary measures	Table 1, Table 2, Table 3
Main results	16	(*a*) Give unadjusted estimates and, if applicable, confounder-adjusted estimates and their precision (eg, 95% confidence interval). Make clear which confounders were adjusted for and why they were included	Table 1, Table 2
		(*b*) Report category boundaries when continuous variables were categorised	Table 1, Table 2
		(*c*) If relevant, consider translating estimates of relative risk into absolute risk for a meaningful time period	NA
Other analyses	17	Report other analyses done—eg analyses of subgroups and interactions, and sensitivity analyses	NA
**Discussion**			
Key results	18	Summarise key results with reference to study objectives	L225 to L234
Limitations	19	Discuss limitations of the study, taking into account sources of potential bias or imprecision. Discuss both direction and magnitude of any potential bias	L327 to L370
Interpretation	20	Give a cautious overall interpretation of results considering objectives, limitations, multiplicity of analyses, results from similar studies, and other relevant evidence	L236 to L324, L723 to L382
Generalisability	21	Discuss the generalisability (external validity) of the study results	L332 to L341
**Other information**			
Funding	22	Give the source of funding and the role of the funders for the present study and, if applicable, for the original study on which the present article is based	L394 to L402

**Note:** An Explanation and Elaboration article discusses each checklist item and gives methodological background and published examples of transparent reporting. The STROBE checklist is best used in conjunction with this article (freely available on the Web sites of PLoS Medicine at http://www.plosmedicine.org/, Annals of Internal Medicine at, http://www.annals.org/and Epidemiology at http://www.epidem.com/). Information on the STROBE Initiative is available at www.strobe-statement.org.

∗Give information separately for exposed and unexposed groups.

**Table:** 

Items		Total population (n = 318)	CALD% (n = 69)	Wider population% (n = 249)	Percentage difference
**Q1. Have you been given information on osteoarthritis from a health professional? % (n)**	Yes	40% (126)	39% (27)	40% (99)	−1%
	No	55% (175)	52% (36)	56% (139)	−4%
	Don't remember	5% (17)	9% (6)	4% (11)	5%
**Q2. Have you been given information about different treatment alternatives? % (n)**	Yes	32% (103)	28% (19)	34% (84)	−6%
	No	64% (204)	64% (44)	64% (160)	0%
	Don't remember	3% (11)	9% (6)	2% (5)	7%
**Q3. Have you been given information about how you can self-manage the disease? % (n)**	Yes	30% (95)	25% (17)	31% (78)	−6%
	No	66% (211)	65% (45)	67% (166)	−2%
	Don't remember	4% (12)	10% (7)	2% (5)	8%
**Q4. Have you been given information about the importance of physical activity and exercise? % (n)**	Yes	56% (178)	55% (38)	56% (140)	−1%
	No	42% (135)	42% (29)	43% (106)	−1%
	Don't remember	2% (5)	3% (2)	1% (3)	2%
**Q5. Have you been referred or offered a referral to a health professional who can advise you about physical activity and exercise? % (n)**	Yes	50% (160)	54% (37)	49% (123)	5%
	No	49% (156)	43% (30)	50% (126)	−7%
	Don't remember	1% (2)	3% (2)	0% (0)	3%
**Q6. Have you been advised to lose weight, if you are overweight? % (n)**	Yes	44% (140)	33% (23)	47% (117)	−14%
	No	20% (65)	16% (11)	22% (54)	−6%
	Not overweight	36% (113)	51% (35)	31% (78)	20%
**Q7. Have you been referred or offered a referral to someone who can help you to lose weight, if you are overweight? % (n)**	Yes	20% (63)	16% (11)	21% (52)	−5%
	No	43% (138)	32% (22)	47% (116)	−15%
	Not overweight	37% (117)	52% (36)	33% (81)	19%
**Q8. If you have problems with daily activities, have these problems been assessed by a health professional? % (n)**	Yes	12% (39)	17% (12)	11% (27)	6%
	No	53% (169)	51% (35)	54% (134)	−3%
	No such problems	35% (110)	32% (22)	35% (88)	−3%
**Q9. If you have problems with walking, has your need for a walking aid been assessed? (e.g. stick, crutch or walker) % (n)**	Yes	12% (39)	22% (15)	10% (24)	12%
	No	47% (150)	42% (29)	49% (121)	−7%
	No such problems	41% (129)	36% (25)	42% (104)	−6%
**Q10. If you have problems related to other daily activities, has your need for appliances and aids been assessed? (e.g.splints, assistive technology for cooking or personal hygiene, a special chair) % (n)**	Yes	10% (31)	15% (10)	8 (21)	7%
	No	48% (152)	46% (32)	48 (120)	−2%
	No such problems	42% (134)	39% (27)	43% (107)	−4%
**Q11. If you have joint pain, has it been assessed by a health professional? % (n)**	Yes	75% (239)	72% (50)	76% (189)	−4%
	No	24% (77)	28% (19)	23% (58)	5%
	No pain	0% (1)	0% (0)	0% (1)	0%
**Q12. If you have joint pain, was paracetamol the first medication that was recommended? % (n)**	Yes	73% (232)	80% (55)	71% (177)	9%
	No	25% (80)	17% (12)	27% (68)	−10%
	No pain	2% (6)	3% (2)	2% (4)	1%
**Q13. If you have prolonged severe joint pain, which is not relieved sufficiently by paracetamol, have you been offered stronger pain killing medication? (e.g co-codamol,codeine, tramadol, co-proxamol, co-dydramol,dihydrocodeine) % (n)**	Yes	39% (124)	38% (26)	39% (98)	−1%
	No	37% (118)	45% (31)	35% (87)	10%
	No prolonged severe pain	24% (76)	17% (12)	26% (64)	−9%
**Q14. If you use anti-inflammatory medications, have you been given information about the effects and possible side-effects of this medication? (e.g ibuprofen (nurofen, Brufen), diclofenac (Voltarol), naproxen (naprosyn), celecoxib (Celebrex)) % (n)**	Yes	36% (116)	36% (25)	37% (91)	−1%
	No	30% (94)	39% (27)	27% (67)	12%
	Not taking such drugs	34% (108)	25% (17)	37% (91)	−12%
**Q15. If you have experienced an acute deterioration of your symptoms, have you been given or offered a steroid injection? % (n)**	Yes	26% (84)	36% (25)	24% (59)	12%
	No	42% (132)	36% (25)	43% (107)	−7%
	Not experienced such deterioration	32% (102)	28% (19)	33% (83)	−5%
**Q16. If you are severely troubled by your osteoarthritis, and exercise and medication do not help, have you been referred or offered a referral for an assessment for operation? (e.g joint replacement) % (n)**	Yes	80% (253)	78% (54)	80% (199)	−2%
	No	9% (28)	10% (7)	8% (21)	2%
	Not severely troubled	11% (37)	12% (8)	12% (29)	0%

**Table:** 

	Country of birth (Total = 38 countries)	Total participants (n = 318)
**Countries not identified as CALD (n** = **6)**	Australia	206
	United States of America	1
	Canada	1
	New Zealand	12
	United Kingdom∗	26
	South Africa	3
**CALD countries (n** = **32)**	Austria	1
	Bulgaria	1
	Chile	1
	Chinaˆ	6
	Cyprus	1
	Egypt	1
	Eritrea	1
	Fiji	1
	Germany	3
	Greece	8
	Hungary	2
	India	5
	Indonesia	3
	Iran	1
	Italy	5
	Latvia	1
	Libya	1
	Malaysia	4
	Malta	1
	Mauritius	2
	Papua New Guinea	1
	Paraguay	1
	Philippines	3
	Poland	2
	Singapore	1
	Somalia	1
	Republic of Korea	1
	Sri Lanka	2
	Turkey	2
	Venezuela	1
	Vietnam	4
	West indies	1

∗United Kingdom includes England, Scotland, Wales and Northern Ireland.

ˆChina includes SARs: Hong Kong and Macau.

**Figure:**
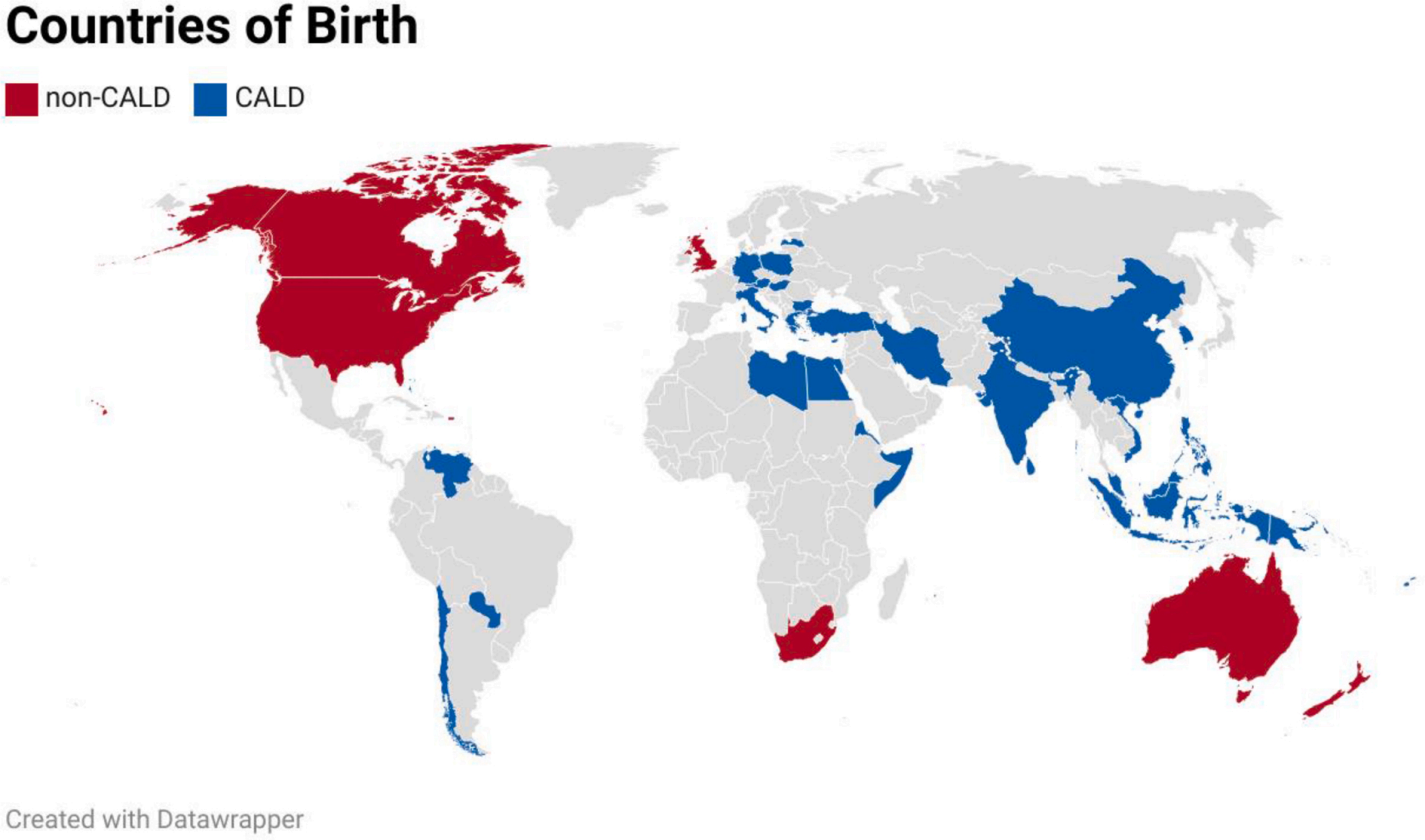


**Table:** 

Languages other than English (n = 24)	Counts
Arabic	2
Bulgarian	1
Cantonese	5
German	3
Greek	6
Hindi	2
Hungarian	1
Italian	4
Korean	1
Malayalam	1
Maltese	2
Mandarin	5
Polish	1
Punjabi	1
Singhalese	1
Somali	1
Spanish	2
Tamil	1
Turkish	4
Vietnamese	4
Farsi	1
Indonesian	1
Tagalog	3
Ukrainian	1
